# Supplementary material for: Determinants of male involvement in antenatal care at Palabek Refugee Settlement, Lamwo district, Northern Uganda
Source: BMC Pregnancy Childbirth. 2023 May 6;23:325. doi: 10.1186/s12884-023-05617-2 (PMC10164295; doi:10.1186/s12884-023-05617-2)
Supplement: Supplementary file 2 — Additional file 2: Questionnaires [file 12884_2023_5617_MOESM2_ESM.docx]

**QUESTIONNAIRE FOR A STUDY ON MALE INVOLVEMENT IN ANC AT PALABEK REFUGEE SETTLEMENT,LAMWO DISTRICT ,NORTHERN UGANDA.**

|  | **PART 1: DETERMINANTS OF MALE INVOLVEMENT IN ANC IN PALABEK REFUGEE SETTLEMENT** | |
| --- | --- | --- |
|  | I**NDIVIDUAL FACTORS** |  |
| 1 | How old is your partner? | --------------------------------------------------- Years |
| 2 | Are you married to your partner? | Yes--------------------------------------------------( )  No---------------------------------------------------( ) |
| 3 | What is your partner’s level of education? | 1.No formal education--------------------( )  2.Attained formal education-------------( ) |
| 4 | How many children do you have together with your partner? | -------------------------------------------------------- |
| 5 | Does your spouse do some work where he earns income? | 1.Yes------------------------------------------------( )  2.No-------------------------------------------------( ) |
| 6 | Does your partner /did your partner have access on ANC information during your pregnancy? | 1.Yes--------------------------------------------- ( )  2.No-----------------------------------------------( ) |
|  | **RELATIONAL FACTORS OF MALE INVOLVEMENT IN ANC** | |
| 7 | Do you/did you have discussions with your spouse about antenatal care during your most recent pregnancy? | 1.Yes ---------------------------------------------- ( )  2No ------------------------------------------------( ) |
| 8 | Are you/Did you live with your partner during your current / most recent pregnancy? | 1.Yes ---------------------------------------------- ( )  2No -------------------------------------------------( ) |
| 9 | Are you/did you live with other family members during your current /most recent pregnancy? | 1.Yes---------------------------------------------- ( )  2No ---------------------------------------------.-( ) |
| 9a | Do family members/did your family members influence your spouse’s participation in antenatal care? (Probe for participation in the different dimensions) | 1.Yes -------------------------------------------- - ( )  2.No ------------------------------------------------( ) |
| 9b | If yes to question above is/was such influence positive or negative | 1.Positive------------------------------------------( )  2.Negative-----------------------------------------( ) |
| 10 | Does peer influence in this community affect how male partners participate in antenatal care? | 1.Yes -------------------------------------------- - ( )  2No -------------------------------------------------( ) |
| 10a | If yes to question above is/was such influence positive or negative | 1.Positive------------------------------------------( )  2.Negative----------------------------------------( ) |
|  | **INSTITUTIONAL FACTORS OF MALE INVOLVEMENT IN ANC** |  |
| 11 | Does the health facility where you access/accessed ANC promote male participation in antenatal care? (Probe for male services, prioritization of men who come for ANC, waiting time etc) | 1.Yes---------------------------------------------- ( )  2.No -----------------------------------------------( ) |
|  | Please mention whether the factors below affect male involvement in ANC in the health facility where you are receiving/received ANC services during the most recent pregnancy. |  |
| 12 | Absence of male health workers | 1.------------------------------------------------- ( )  2.No --------------------------------------------( ) |
| 13 | No privacy/ male rooms | 1.Yes---------------------------------------------- ( )  2.No -----------------------------------------------( ) |
| 14 | Long waiting hours | 1.Yes--------------------------------------------- ( )  2.No ----------------------------------------------( ) |
| 15 | Fear of HIV test | 1.Yes---------------------------------------------.( )  2.No -----------------------------------------------( ) |
| 16 | Attitude of health worker | 1.Yes---------------------------------------------- ( )  2.No -----------------------------------------------( ) |
| 17 | On average, how far is your husband’s most frequented place to the nearest health facility where you are accessing/accessed ANC services from during your current/previous pregnancy? | 1.Less than 3km-----------------------------------( )  2.More than 3km----------------------------------( ) |
|  | **INSTITUTIONAL FACTORS OF MALE INVOLVEMENT IN ANC** |  |
| 18 | Do cultural gender norms in the community affect male participation in antenatal care? (Probe for aspects of cultural gender norms) | 1.Yes -----------------------------------------------( )  2No---------.--------------------------------------( ) |
| 19 | Are there community structures/plat forms that promote male involvement in antenatal care in your community | 1.Yes ----------------------------------------------( )  2No-------------------------------------------------( ) |
| 20 | Do you know about /have you have heard of any government/health facility initiatives regarding male participation in antenatal care? | 1.Yes ---------------------------------------------- ( )  2No------------------------------------------------ ( ) |
|  | **PART 2: PREVALENCE OF MALE INVOLVEMNT IN ANTENATAL CARE** | |
| 21 | Does/Did your partner make joint plans for your pregnancy together with you? | 1.Yes------------------------------------------------( )  2.No-------------------------------------------------( ) |
| 22 | Does/Did your partner attend ANC with you during your current/most recent pregnancy? | 1.Yes-----------------------------------------------( )  2No-------------------------------------------------( ) |
| 23 | Does/Did your partner provide you with some money for food, transport, maternity clothes etc. during your current/most recent pregnancy? | 1.Yes------------------------------------------------( )  2.No-------------------------------------------------( ) |
| 24 | Does/Did your partner support in household daily work like washing plates, cleaning the house, cleaning the compound, or washing clothes during your current/most recent pregnancy? | 3.Yes------------------------------------------------( )  4No-------------------------------------------------( ) |
| 25 | Does /Did your partner get involved in discussing issues of ANC with you during your current/most recent pregnancy? | 1.Yes------------------------------------------------( )  2No-------------------------------------------------( ) |
| 26 | Does/Did your partner get involved in discussing your pregnancy with your health care provider during your current/most recent pregnancy? | 1.Yes-----------------------------------------------( )  2.No-------------------------------------------------( ) |

Instructions: Please tick the most appropriate response/fill in the blank spaces where applicable
